# Genome-wide identification and investigation of monosaccharide transporter gene family based on their evolution and expression analysis under abiotic stress and hormone treatments in maize (*Zea mays* L.)

**DOI:** 10.1186/s12870-024-05186-2

**Published:** 2024-06-04

**Authors:** Jialun Zhu, Tianfeng Li, Jing Ma, Wenyu Li, Hanyu Zhang, Tsyganova Nadezhda, Yanshu Zhu, Xiaomei Dong, Cong Li, Jinjuan Fan

**Affiliations:** 1 College of Bioscience and Biotechnology, Shenyang Agricultural University, Shenyang, Liaoning 110866 China; 2 Saint-Petersburg State Agrarian University, Peterburgskoe shosse, Pushkin, St. Petersburg, 196601 Russia; 3 Shenyang City Key Laboratory of Maize Genomic Selection Breeding, Shenyang, Liaoning 110866 China

**Keywords:** Genome-wide identification, *Zea mays* L., Monosaccharide transporter (MST), Gene family, Abiotic stress

## Abstract

**Background:**

Monosaccharide transporter (MST) family, as a carrier for monosaccharide transport, plays an important role in carbon partitioning and widely involves in plant growth and development, stress response, and signaling transduction. However, little information on the MST family genes is reported in maize (*Zea mays*), especially in response to abiotic stresses. In this study, the genome-wide identification of MST family genes was performed in maize.

**Result:**

A total of sixty-six putative members of MST gene family were identified and divided into seven subfamilies (including SPT, PMT, VGT, INT, pGlcT, TMT, and ERD) using bioinformatics approaches, and gene information, phylogenetic tree, chromosomal location, gene structure, motif composition, and *cis*-acting elements were investigated. Eight tandem and twelve segmental duplication events were identified, which played an important role in the expansion of the ZmMST family. Synteny analysis revealed the evolutionary features of *MST* genes in three gramineous crop species. The expression analysis indicated that most of the PMT, VGT, and ERD subfamilies members responded to osmotic and cadmium stresses, and some of them were regulated by ABA signaling, while only a few members of other subfamilies responded to stresses. In addition, only five genes were induced by NaCl stress in MST family.

**Conclusion:**

These results serve to understand the evolutionary relationships of the ZmMST family genes and supply some insight into the processes of monosaccharide transport and carbon partitioning on the balance between plant growth and development and stress response in maize.

**Supplementary Information:**

The online version contains supplementary material available at 10.1186/s12870-024-05186-2.

## Background

The industrial activities of human caused the trends of climate warming and constant changes in the natural environment [1, 2]. Many abiotic stresses such as drought, salinity, low temperature, and heavy metals pollution affect the growth and development of plants and the yield of crops, and further threaten food security and human health [3]. Sugars are the main form of long-distance transportation and distribution of photosynthate and play an important role in plant growth and development [4]. Sugar not only constitutes metabolites, nutrients, and signal molecules, but also can be used as osmotic substances. One way that plants protect cell structure and alleviate the damage of abiotic stress is by accumulating osmotic substances or compatible solutes [4, 5]. In *Arabidopsis thaliana*, sugar plays an important role in protecting plant structure by increasing the concentration of soluble sugars (sucrose, glucose and fructose) in sink leaves and sucrose in phloem sap, maintaining water potential and improving plant stress tolerance [6–8]. Therefore, sugar transport and distribution are key for crops in yielding and coping with abiotic stress [9, 10].

In plants, various sugar transporters are involved in the transport of sugar from source to sink, of which the major facilitator superfamily (MFS) is an important member [11]. MFS can be generally divided into the sucrose transporter (SUT) family and MST family according to the different transport substrates. MSTs are complete membrane proteins that can participate in the transmembrane transport of monosaccharides [12]. The MST family can also be further divided into seven subfamilies based on their substrate specificities and sequence features, including Sugar Transport Protein (STP), Polyol/Monosaccharide Transporter (PMT), Vacuolar Glucose Transporter (VGT), Inositol Transporter (INT), Plastidic Glucose Transporter (pGlcT), Tonoplast Membrane Transporter (TMT), and Early-Responsive to Dehydration six-like (ERD) [13].

STP is a sugar transporter that mainly transports hexose. In Arabidopsis, most STPs exhibited a broad spectrum of absorption characteristics of substrates. AtSTP1 is involved in Arabidopsis germination and root development as a transporter protein for sugar alcohols [14]. In *atstp1* mutant, the ability to transport sugar alcohols (D-glucose, D-galactose, and D-mannose) is greatly weakened compared with the wild type [14]. By investigating *AtSTP6*-promoter::*GUS* plants and conducting in situ hybridization experiments at the late stage of pollen development, it is determined that *AtSTP6* was expressed. A transposon-tagged Arabidopsis mutant also demonstrates that the *atstp6* mutation may have an impact on pollen vitality, pollen germination, fertilization, and seed production [15]. PMT proteins (PMTs) not only transport mannitol, sorbitol, xylitol, and other polyols, but also transport monosaccharides. The first PMT gene is found in *Apium graveolens* L., named *AgMAT1*, and it plays an important role in the loading process of phloem mannitol [16]. Arabidopsis genome contains six PMT subfamily genes, named *AtPMT1* to *AtPMT6*. *AtPMT5* is located in the plasma membrane and can transport pentoses such as sugar alcohols (sorbitol, xylitol, erythritol, and glycerol), hexose, and ribose. *AtPMT5* is highly expressed in Arabidopsis roots and plays an important role in plant morphological construction [17]. In plants, the VGT subfamily generally has only 2–3 members, and plays a key role in the process of seed germination, flowering, and other growth and development [18, 19]. In Arabidopsis, AtVGT1 is H^+^/glucose reverse transporter located on the vacuolar plastid, which is involved in the transport and storage of monosaccharides in vacuoles. AtVGT1 also plays a key role in the process of seed germination, flowering, and other growth and development, and the *atvgt1* mutant has a lower germination rate and delays flowering [20]. INT proteins are highly specific H^+^-inositol symporters, and the functions of them are in the transportation and distribution of inositol. Four *INT* genes (*AtINT1*-*4*) are identified in Arabidopsis, of which *AtINT4* is the first identified INT subfamily member. *AtINT4* is highly expressed in Arabidopsis pollen and phloem companion tissue, which is related to plant pollen development and participated in inositol loading in phloem [21]. pGlcT proteins (pGlcTs) can transport glucose. The pGlcT gene is cloned in spinach (*Spinacia oleracea* L.) and proves the role in transporting starch and hydrolyzing glucose [21]. In Arabidopsis, the *atpglct1*, *atpglct2*, and *atpglct1*/*atpglct2* mutants show growth and development inhibition in varying degrees [22]. TMT proteins (TMTs, also known as ATZ) are also localized on vacuolar plastids. Previous studies reported that TMTs are involved in the transport of sucrose on vacuoles. In the *Attmt1*/*Attmt2* double mutant, the sucrose transportation to vacuoles is impaired [23]. The majority of the ERD6-like protein is found on the vacuolar membrane and is in charge of the transmembrane transport of sugar in the vacuoles [24]. In Arabidopsis, ERD (also known as SFPs) is the largest subfamily in the MST family and responds to abiotic stresses. For instance, *AtERD1* and *AtERD2* also respond to different stress treatments, and they may function coordinately with the vacuolar invertase to regulate osmotic pressure by affecting the accumulation of sugar in plant cells [25]. Genome-wide identification of the MST family has been performed in many plants, such as Arabidopsis, rice, grape, and tobacco [12, 26–28].

The distribution of monosaccharide transporters in maize plants directly affects plant growth and development and abiotic stress response. However, genome-wide identification, evolutionary analysis, and response to abiotic stresses of the maize monosaccharide transporter family have not yet been reported. In this study, 66 *MST* genes were identified from the maize genome. Phylogenetic analysis, gene structure analysis, and synteny analysis were performed to understand the evolution and amplification of the ZmMST family, and the expression analysis in different tissues and under different treatments was performed to explore the response to abiotic stresses. These results provide insights into the evolution of the maize MST family and their role in maize growth and development and abiotic stress response. The identification and characterization of *ZmMST* genes may provide opportunities for the optimization of maize variety selection and breeding.

## Results

### Identification and phylogenetic analysis of the MST gene family members in maize

The Arabidopsis MST proteins were queried against the maize genome using BLASTP to search for maize *MST* genes. The gene domain was manually confirmed through the NCBI CDD, SMART, and Pfam websites, and the protein sequence length (number of amino acids), molecular weight, and isoelectric point were determined by the ExPASy proteomics system. The sequences of the monosaccharide transporter family revealed by screening have conserved structural domains. Finally, 66 complete monosaccharide transporter sequences were identified and divided into seven subfamilies in maize, including STP (23 members), PMT (20 members), VGT (2 members), INT (4 members), pGlcT (4 members), TMT (4 members), and ERD (9 members) subfamilies. Gene names, AA accession number, length of the gene, amino acid numbers, molecular weights, chromosomal locations, and pIs were listed in Table 1. Meanwhile, 64, 69, and 77 genes of the MST family were identified in rice, sorghum, and millet, respectively, as shown in Additional file 1.

**Table 1:** The identification of MST members in maize

Gene name	AA accession number	Length of gene(bp)	Number of amino acid(aa)	Molecular weight (Da)	Theoretical(pI)	Chr	strand Transcript	
***ZmSTP1***	***Zm00001d027268***	**1578**	**525**	**57,494.9**	**9.26**	**Chr1:103477Chr7:1,037,365**	**-﻿**	**1**
***ZmSTP2***	***Zm00001d028230***	**1545**	**514**	**56,685**	**9.12**	**Chr1:27,307,350:27,313,346**	**-**	**1**
***ZmSTP3***	***Zm00001d032409***	**1611**	**536**	**56,846.1**	**9.44**	**Chr1:22583003Chr1:225,834,001**	**-**	**1**
***ZmSTP4***	***Zm00001d032906***	**1527**	**508**	**54,319**	**9.19**	**Chr1:24270208Chr1:242,703,904**	** + **	**1**
***ZmSTP5***	***Zm00001d003468***	**1131**	**376**	**39,731.1**	**9.92**	**Chr2:4523420Chr6:45,238,299**	** + **	**1**
***ZmSTP6***	***Zm00001d003469***	**1542**	**513**	**54,767.7**	**9.04**	**Chr2:4523873Chr8:45,242,608**	**-**	**2**
***ZmSTP7***	***Zm00001d003471***	**1674**	**557**	**59,538.2**	**10.18**	**Chr2:4528734Chr1:45,292,164**	**-**	**1**
***ZmSTP8***	***Zm00001d005594***	**1557**	**518**	**56,633**	**9.23**	**Chr2:18012113Chr9:180,123,254**	** + **	**1**
***ZmSTP9***	***Zm00001d007078***	**1473**	**490**	**53,551.9**	**9.61**	**Chr2:221,769,030:221,780,225**	** + **	**1**
***ZmSTP10***	***Zm00001d044245***	**1530**	**509**	**55,644.6**	**9.88**	**Chr3:22292368Chr9:222,925,949**	**-**	**1**
***ZmSTP11***	***Zm00001d049467***	**1545**	**514**	**55,430.5**	**7.72**	**Chr4:3137898Chr1:31,380,525**	**-**	**1**
***ZmSTP12***	***Zm00001d050860***	**1566**	**521**	**56,348.6**	**9.29**	**Chr4:12723237Chr2:127,244,717**	** + **	**1**
***ZmSTP13***	***Zm00001d053846***	**1656**	**551**	**59,924.1**	**8.75**	**Chr4:24217007Chr3:242,172,587**	**-**	**2**
***ZmSTP14***	***Zm00001d016919***	**1341**	**447**	**48,442.5**	**9.85**	**Chr5:18005825Chr7:180,059,691**	** + **	**1**
***ZmSTP15***	***Zm00001d018627***	**1572**	**523**	**57,035.4**	**9.67**	**Chr7:13357Chr10:1,339,124**	**-**	**1**
***ZmSTP16***	***Zm00001d019138***	**1542**	**513**	**56,137.9**	**8.85**	**Chr7:1871972Chr8:18,721,630**	**-**	**1**
***ZmSTP17***	***Zm00001d020071***	**1569**	**522**	**57,267.6**	**9.6**	**Chr7:9039738Chr4:90,401,326**	** + **	**1**
***ZmSTP18***	***Zm00001d020463***	**1572**	**523**	**7884**	**9.19**	**Chr7:11645175Chr9:116,453,072**	** + **	**2**
***ZmSTP19***	***Zm00001d021775***	**1575**	**524**	**56,912.7**	**9.25**	**Chr7:16284589Chr1:162,848,484**	**-**	**1**
***ZmSTP20***	***Zm00001d045395***	**1557**	**518**	**54,517.2**	**10.13**	**Chr9:2053261Chr2:20,535,038**	** + **	**1**
***ZmSTP21***	***Zm00001d025572***	**1524**	**507**	**53,521.9**	**9.46**	**Chr10:12264185Chr7:122,645,133**	**-**	**1**
***ZmSTP22***	***Zm00001d025573***	**1371**	**456**	**49,861.4**	**10.02**	**Chr10:12270886Chr5:122,710,678**	**-**	**1**
***ZmSTP23***	***Zm00001d000183***	**1563**	**520**	**56,829.3**	**9.58**	**ctg18Chr1:30749Chr1:312,293**	** + **	**1**
***ZmPMT1***	***Zm00001d028144***	**1578**	**525**	**56,032.4**	**8.05**	**Chr1:2401225Chr4:24,014,074**	**-**	**1**
***ZmPMT2***	***Zm00001d028151***	**1575**	**524**	**56,153.1**	**9.23**	**Chr1:2429641Chr3:24,298,623**	**-**	**4**
***ZmPMT3***	***Zm00001d029645***	**1560**	**519**	**55,295.4**	**9.91**	**Chr1:8094488Chr6:80,947,089**	** + **	**1**
***ZmPMT4***	***Zm00001d030464***	**1527**	**508**	**54,026.8**	**9.3**	**Chr1:13452100Chr8:134,522,680**	** + **	**1**
***ZmPMT5***	***Zm00001d001817***	**1617**	**538**	**55,880.6**	**7.13**	**Chr2:142570Chr1:1,428,025**	**-**	**1**
***ZmPMT6***	***Zm00001d001818***	**1650**	**549**	**57,360.9**	**7.13**	**Chr2:143207Chr7:1,434,514**	**-**	**1**
***ZmPMT7***	***Zm00001d002864***	**1602**	**533**	**57,519.7**	**6.53**	**Chr2:2496079Chr7:24,963,400**	** + **	**1**
***ZmPMT8***	***Zm00001d006688***	**1551**	**516**	**54,367.5**	**9.39**	**Chr2:21447246Chr4:214,474,594**	** + **	**1**
***ZmPMT9***	***Zm00001d006697***	**1563**	**520**	**55,079.1**	**9.37**	**Chr2:21453545Chr7:214,537,722**	**-**	**1**
***ZmPMT10***	***Zm00001d048771***	**1446**	**481**	**50,201.4**	**8.55**	**Chr4:52585Chr10:5,260,332**	** + **	**1**
***ZmPMT11***	***Zm00001d048774***	**1506**	**501**	**52,136.4**	**9.3**	**Chr4:528403Chr7:5,285,944**	** + **	**1**
***ZmPMT12***	***Zm00001d048775***	**1350**	**449**	**46,171.2**	**9.63**	**Chr4:532866Chr1:5,330,696**	** + **	**1**
***ZmPMT13***	***Zm00001d048776***	**1464**	**487**	**50,935.3**	**8.71**	**Chr4:538077Chr9:5,382,734**	** + **	**1**
***ZmPMT14***	***Zm00001d021935***	**1542**	**513**	**54,830.1**	**9.75**	**Chr7:16637624Chr6:166,378,812**	** + **	**2**
***ZmPMT15***	***Zm00001d021936***	**1533**	**510**	**53,894**	**8.37**	**Chr7:16638310Chr8:166,385,301**	** + **	**2**
***ZmPMT16***	***Zm00001d021938***	**1542**	**513**	**54,255.2**	**9.09**	**Chr7:16655059Chr1:166,552,978**	**-**	**1**
***ZmPMT17***	***Zm00001d021942***	**1485**	**495**	**52,501.1**	**8.86**	**Chr7:16660746Chr4:166,609,263**	**-**	**1**
***ZmPMT18***	***Zm00001d048178***	**1596**	**531**	**56,736.5**	**8.86**	**Chr9:15171082Chr9:151,713,323**	**-**	**5**
***ZmPMT19***	***Zm00001d023939***	**1437**	**478**	**50,163.6**	**8.9**	**Chr10:3031494Chr4:30,317,071**	** + **	**2**
***ZmPMT20***	***Zm00001d023941***	**1470**	**489**	**50,575.4**	**8.55**	**Chr10:30,402,000:30,405,639**	** + **	**2**
***ZmVGT1***	***Zm00001d012938***	**1398**	**465**	**49,263.3**	**8.75**	**Chr5:223036Chr5:2,234,904**	**-**	**4**
***ZmVGT2***	***Zm00001d014435***	**1554**	**517**	**55,327**	**5.77**	**Chr5:4670830Chr3:46,712,407**	** + **	**8**
***ZmINT1***	***Zm00001d018803***	**1758**	**585**	**62,441.4**	**8.58**	**Chr7:567092Chr8:5,673,511**	** + **	**1**
***ZmINT2***	***Zm00001d019537***	**1458**	**485**	**51,911.3**	**8.89**	**Chr7:41,043,850:41,045,307**	**-**	**1**
***ZmINT3***	***Zm00001d025749***	**1602**	**533**	**56,679.5**	**5.92**	**Chr10:12828174Chr3:128,287,246**	**-**	**2**
***ZmINT4***	***Zm00001d025834***	**1746**	**581**	**62,805.8**	**8.44**	**Chr10:13139539Chr4:131,399,111**	**-**	**2**
***ZmpGlcT1***	***Zm00001d039973***	**1620**	**539**	**56,585.5**	**9.11**	**Chr3:2131176Chr2:21,317,041**	** + **	**11**
***ZmpGlcT2***	***Zm00001d053334***	**1488**	**495**	**58,078**	**6.71**	**Chr4:22658326Chr6:226,590,664**	**-**	**1**
***ZmpGlcT3***	***Zm00001d020374***	**1653**	**550**	**56,806.8**	**9.36**	**Chr7:11002322Chr5:110,030,221**	** + **	**10**
***ZmpGlcT4***	***Zm00001d008567***	**1620**	**539**	**53,360.3**	**8.31**	**Chr8:1314614Chr1:13,153,169**	** + **	**11**
***ZmTMT1***	***Zm00001d029762***	**1305**	**434**	**60,523.5**	**4.96**	**Chr1:8613001Chr4:86,134,527**	** + **	**13**
***ZmTMT2***	***Zm00001d048823***	**1125**	**374**	**59,874**	**6.46**	**Chr4:601307Chr3:6,018,723**	**-**	**7**
***ZmTMT3***	***Zm00001d014872***	**2292**	**763**	**80,956.6**	**4.46**	**Chr5:6660522Chr8:66,608,036**	**-**	**3**
***ZmTMT4***	***Zm00001d016274***	**1806**	**601**	**68,874**	**5.73**	**Chr5:15513335Chr4:155,138,132**	**-**	**11**
***ZmERD1***	***Zm00001d029254***	**1533**	**510**	**51,871.2**	**5.74**	**Chr1:6385984Chr5:63,864,152**	**-**	**2**
***ZmERD2***	***Zm00001d040243***	**1452**	**483**	**39,799.5**	**8.96**	**Chr3:3341091Chr8:33,414,667**	**-**	**4**
***ZmERD3***	***Zm00001d039051***	**1503**	**500**	**52,615.2**	**9.25**	**Chr6:16929398Chr5:169,298,831**	** + **	**9**
***ZmERD4***	***Zm00001d039052***	**1233**	**410**	**53,240.9**	**9.6**	**Chr6:16932079Chr3:169,327,039**	** + **	**1**
***ZmERD5***	***Zm00001d008374***	**1470**	**489**	**31,835.5**	**10.37**	**Chr8:669913Chr9:6,703,444**	** + **	**1**
***ZmERD6***	***Zm00001d009600***	**1125**	**374**	**54,327.8**	**7.31**	**Chr8:7249771Chr8:72,504,565**	** + **	**36**
***ZmERD7***	***Zm00001d009603***	**1491**	**496**	**53,739.2**	**8.44**	**Chr8:72763170Chr8:72,767,938**	** + **	**4**
***ZmERD8***	***Zm00001d009605***	**1497**	**498**	**44,025.2**	**9.34**	**Chr8:7286116Chr4:72,868,595**	** + **	**2**
***ZmERD9***	***Zm00001d009669***	**1521**	**506**	**51,148.7**	**8.31**	**Chr8:7514623Chr3:75,149,733**	**-**	**10**

**Fig. 1:**
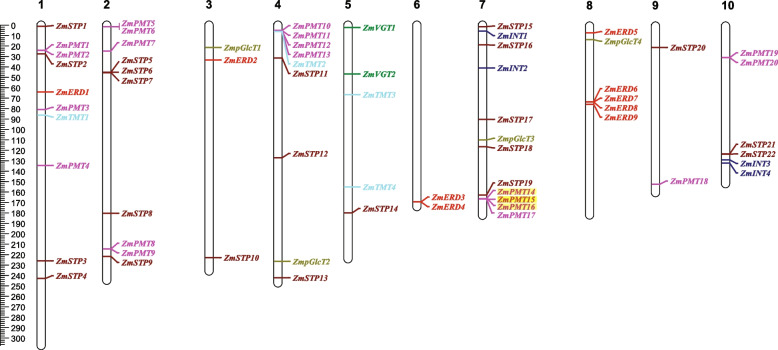
Chromosomal distribution of *ZmMST* genes. *ZmPMT* genes, *ZmpGlcT* genes, *ZmERD* genes, *ZmSTP* genes, *ZmINT* genes, *ZmVGT* genes, and *ZmTMT* genes are highlighted in purple, yellow-green, red, dark brown, dark blue, dark green, and light blue, respectively. Tandem repeat genes are highlighted in yellow

As shown in Fig. 1, the *MST* genes were randomly distributed on 10 maize chromosomes. Among them, 12 *MST* genes were distributed on chromosome (chr) 7, and only two genes existed on chr 6 and 9. The STP subfamily genes were dispersed across 8 of the 10 chromosomes, and *ZmPMT* genes were dispersed across 6 of the 10 chromosomes. *ZmVGT* genes had only two genes and were located on chr 5. *ZmTMT* genes were located on chr 1, 4, and 5. *ZmINT* genes were located on chr 7 and 10, respectively. *ZmpGlcT* genes were located on chr 3, 4, 7, and 8, respectively. *ZmERD* genes were located on chr 1, 3, and 8.

**Fig. 2:**
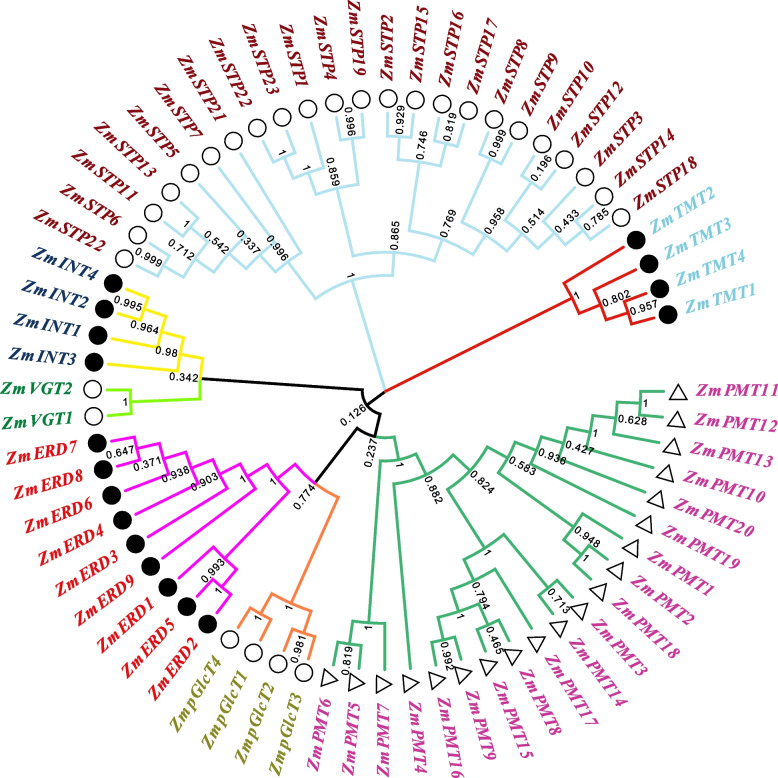
Phylogenetic tree for MST proteins of *Zea mays*. Multiple sequence alignment of the MST domains was performed using MUSCLE, and the phylogenetic tree was constructed using MEGA 7.0 with the maximum likelihood method with 1000 bootstrap replicates. Proteins of ZmPMT, ZmpGlcT, ZmERD, ZmSTP, ZmINT, ZmVGT, and ZmTMT are highlighted in purple, yellow-green, red, dark brown, dark blue, dark green, and light blue, respectively

**Fig. 3:**
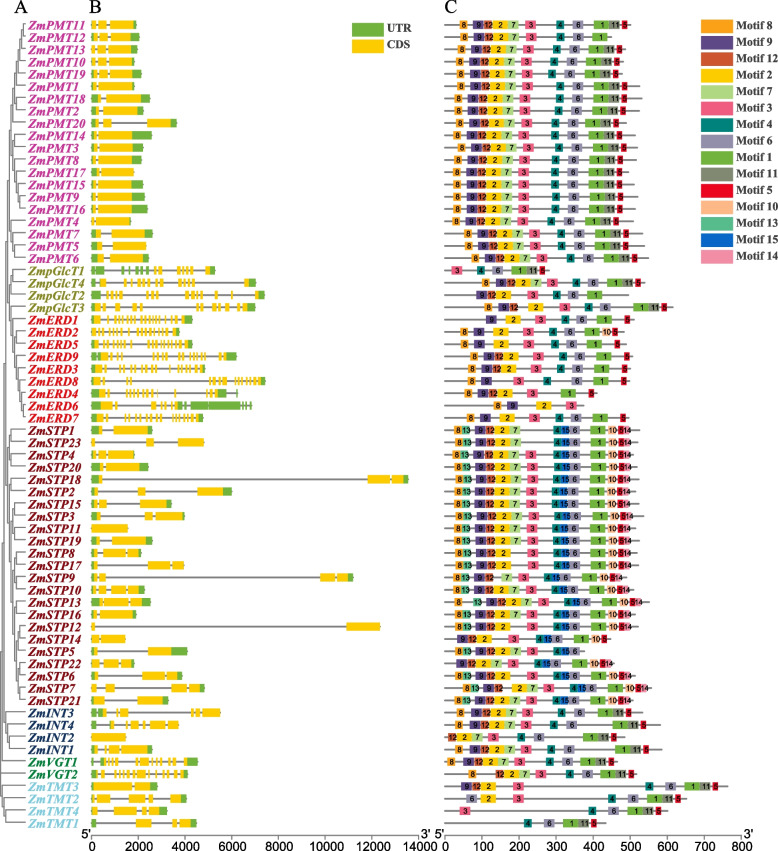
Phylogenetic relationships, gene structure, and conserved motif analyses of *ZmMST* genes based on phylogenetic relationships. All motifs were identified with the MEME Suite using the complete amino acid sequences. Exon–intron structure analyses were performed with TBtools. **A** Neighbor-joining tree indicating evolutionary relationships. **B** Exon–intron structure. Green boxes, yellow boxes, and black lines indicate the untranslated region, coding sequence, and gene length, respectively. **C** Conserved motifs

To explore the systematic evolution, a phylogenetic tree of MST family genes in maize was constructed. As shown in Figs. 2 and 3A, *ZmMST* genes were divided into five main branches and seven groups, which was consistent with the results of existing studies. The VGT subfamily and INT subfamily possessed a closed relationship, while the relationship of ERD and pGlcT was close. Meanwhile, phylogenetic trees were constructed in seven subfamilies, including members from three species of maize, rice, and Arabidopsis (Additional file 2–8).

### Gene structure and motif composition of the maize MST gene family

The exon–intron organizations of the MST family genes were detected to comprehend the evolution of the MST family in maize. As shown in Fig. 3B, *ZmSTP* genes possessed one to four exons (20 with two to three exons, 3 with four exons, and *ZmSTP11* with only one exon). Although the ZmPMT subfamily has many genes, the gene structure tends to be conserved, and all *ZmPMT* genes possess two to three exons (14 members with two exons and 6 members with three exons). The ZmVGT subfamily contains only two genes, *ZmVGT1* possesses twelve exons and *ZmVGT2* possesses fourteen exons. *ZmINT* and *ZmTMT* genes possesses one to six exons. *ZmpGlcT* genes possess 12 to 14 exons. The ZmERD subfamily is different from other subfamilies, which contain an extensive number of exons, from 7 to 18.

The conserved motifs of MST proteins were identified by the MEME motif program. The result was shown in Fig. 3C. Almost all MST family members contained six conserved motifs, motifs 1 to 6, and each subfamily contained different conserved motifs. Except for the ERD and TMT subfamilies, the other five subfamilies all contain motif 7. Motif 8 exists in almost all subfamilies except the TMT and motif 11 exists in all subfamilies except the ERD and STP. While motifs 10, 13, 14, and 15 only exist in the STP subfamily. Overall, the gene structures and conserved motif compositions of MSTs were similar in the same subfamily.

### Synteny analysis of *MST* genes

**Fig. 4:**
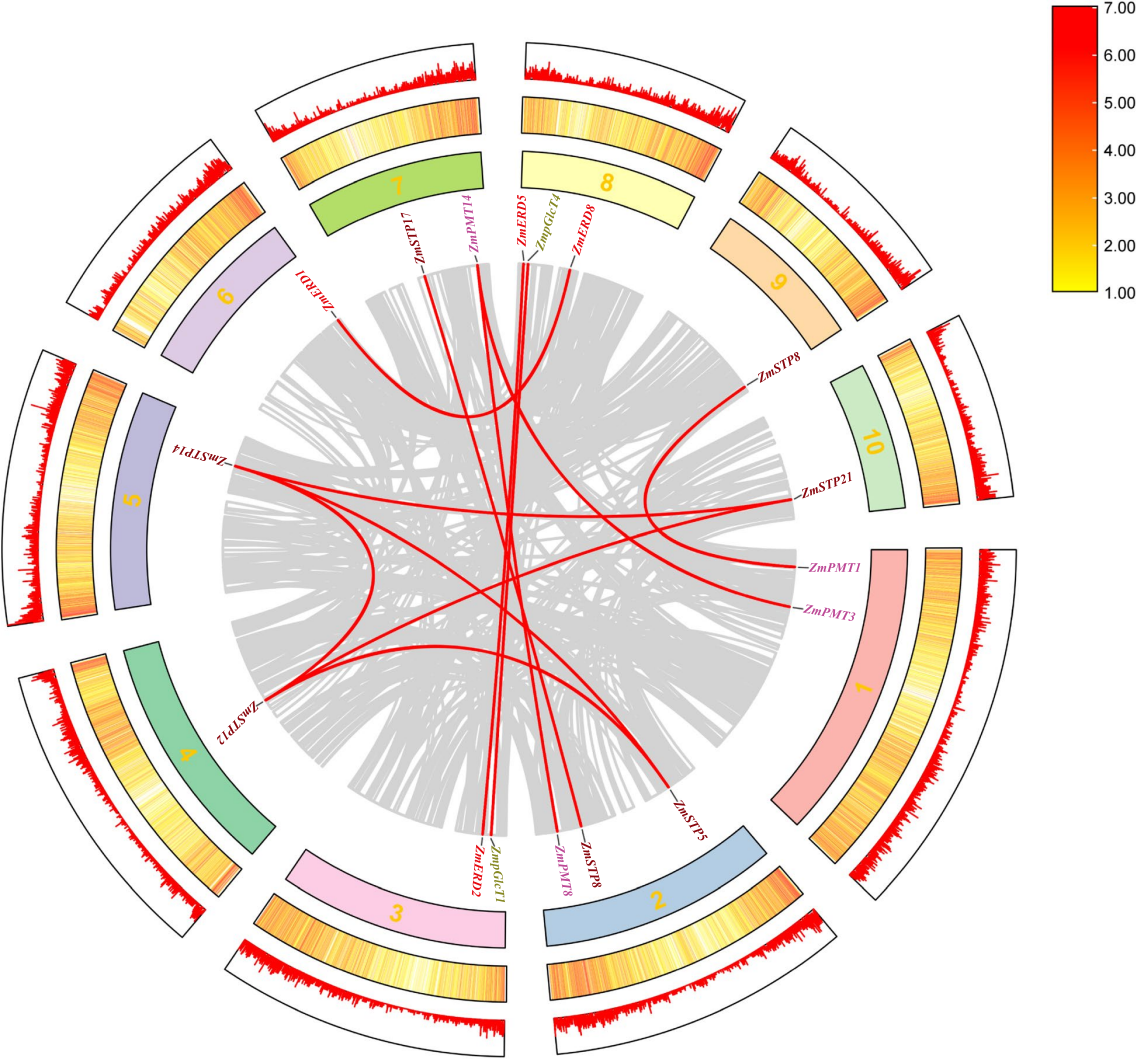
Synteny analysis of the MST family in maize. The two outermost circles are the density of the maize genome, the third circle is the numbering of maize chromosomes, the fourth circle is the ID of genes with segmental repeat relationships, and the innermost circle is the result of the analysis of covariance within the maize genome. Red curves linking *ZmMST* genes indicate duplicated gene pairs in the ZmMST family

To analyze the expansion of the MST family, gene duplication events, both tandem duplication (TD) and segmental duplication (SD) were detected. Eight pairs of tandem replication gene pairs (Fig. 1), including *ZmSTP5*/*6*, *ZmSTP21*/*22*, *ZmPMT1*/*2*, *ZmPMT2*/*3*, *ZmPMT5*/*6*, *ZmPMT14*/*15*, *ZmPMT15*/*16* and *ZmERD3*/*4*, and 12 segmental duplication events, including *ZmSTP5*/*13*, *ZmSTP6*/*15*, *ZmSTP9*/*18*, *ZmSTP13*/*15*, *ZmSTP15*/*22*, *ZmSTP13*/*22*, *ZmPMT1*/*8*, *ZmPMT3*/*4*, *ZmPMT8*/*14*, *ZmpGlcT1*/*4*, *ZmERD2*/*5*, and *ZmERD3*/*6* were identified in maize (Fig. 4).

**Fig. 5:**
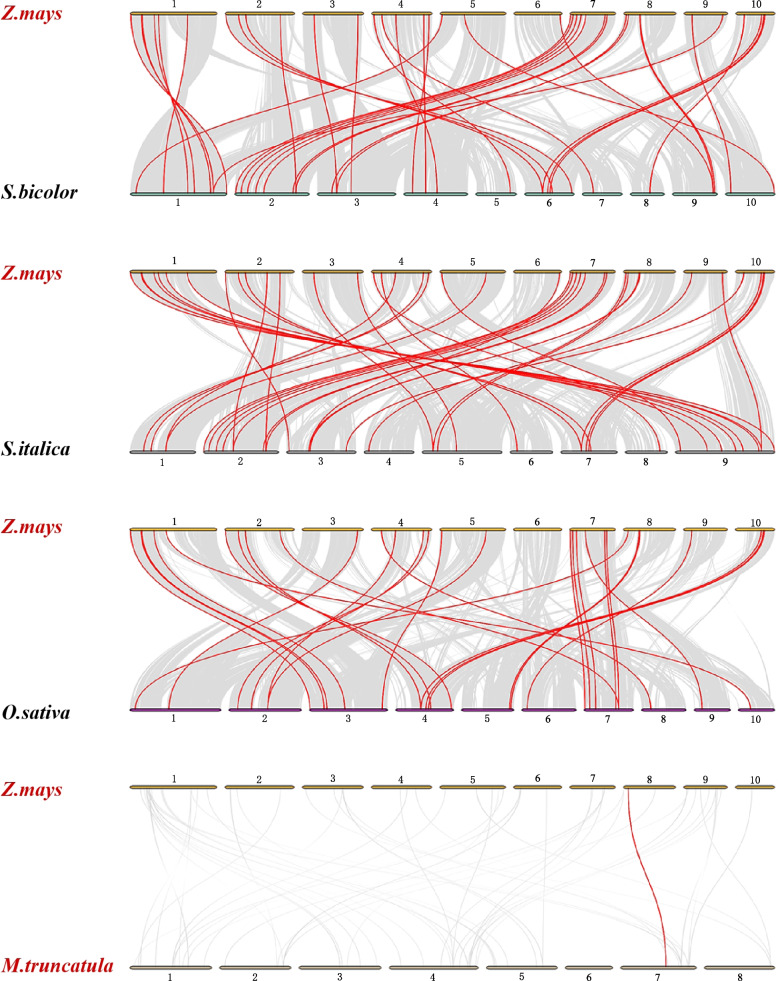
Synteny analysis of *MST* genes between *Zea mays* (*Z*.*mays*) and *Sorghum bicolor* (*S*.*bicolor*), *Setaria italica* (*S*.*italica*), *Oryza sativa* (*O*.*sativa*), and *Medicago truncatula* (*M*.*truncatula*), respectively. Gray lines in the background show collinear blocks in the genomes of maize and other four plants*,* and red lines highlight the collinear MST gene pairs

To further explore the evolutionary mechanisms of the MST family, the syntenic maps of maize were constructed and associated with four representative plant species, including the monocotyledons *Sorghum bicolor*, *Setaria italica*, *Oryza sativa*, and the dicotyledon *Medicago truncatula* (Fig. 5). A total of 41 MST family genes in maize showed syntenic relationships with those in millet, followed by sorghum (47) and rice (33), and there was only one pair of homologous genes in alfalfa (on chr 8), and specific gene pairs are shown in Additional file 9.

To better understand the evolutionary constraints acting on the MST gene family, the Ka/Ks ratios of the tandem and segmental duplications in MST gene pairs were calculated (Additional file 10). Five pairs of genes had Ka/Ks > 1 (*ZmSTP5*/*6*, *ZmSTP5*/*12*, *ZmERD2*/*5*, *ZmERD3*/*4*, and *ZmERD3*/*6*). The Ka/Ks of others are all less than 1.

### Analysis of the *cis*-acting element in MST gene promoter regions

**Fig. 6:**
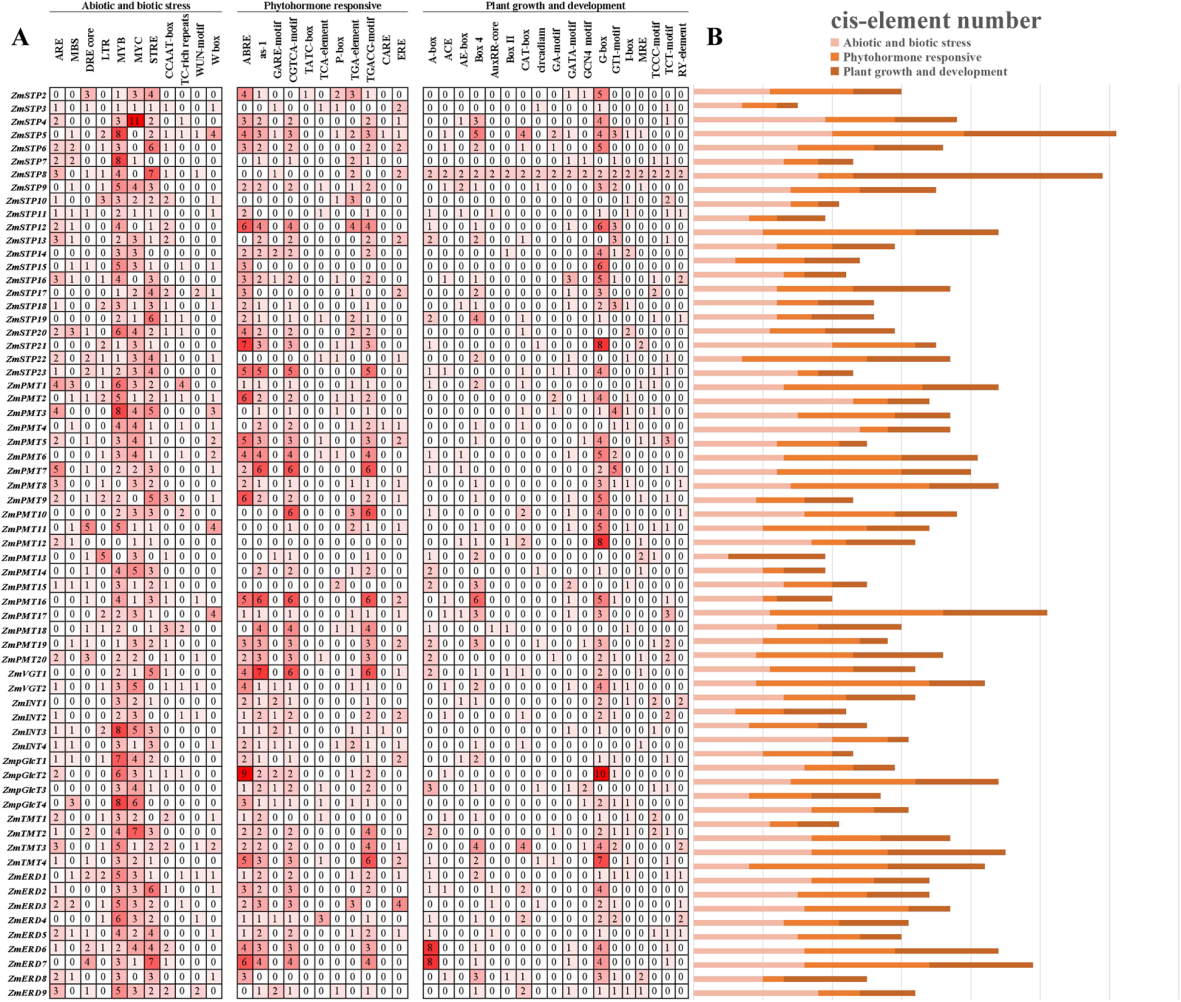
*Cis*-acting elements in the ZmMST gene family. **A** Number of different elements in the promoter region of the *ZmMST* genes, as indicated by different color intensities and numbers in the grid. **B** Total number of *cis*-acting elements in each response category. Sandy-brown indicates abiotic and biotic stresses, goldenrod indicates phytohormones, and chocolate represents plant growth and development

To explore the potential regulatory mechanisms of MST family genes, the 1.5 kb upstream promoter region of *MST* genes was submitted into Plant CARE to detect the conserved *cis*-elements. Then, the identified *cis*-acting elements were divided into three categories: abiotic and biotic stress, phytohormone responsive, and plant growth and development (Fig. 6).

In the categories of abiotic and biotic stresses, MYB (CCAAT-box) and MYC (CACAT-box) were the two types of *cis*-elements with the largest proportion in the MST family. Meanwhile, the anaerobic response element ARE, drought response element DRE, and low-temperature element LTR were also widely distributed in the promoter regions of *MST* genes. For phytohormone responsive elements, the ABRE, TCA, as-1, and CGTCA-motif which are involved in Abscisic Acid (ABA), salicylic acid (SA), and methyl jasmonate (MeJA) responses respectively were detected in the most of MST gene promoter regions. In addition, 18 *cis*-elements related to plant growth and development were identified, most of which were involved in the light response, accounting for more than half of the group.

### Expression analysis of *MST* genes in different maize tissues and in response to different treatments

**Fig. 7:**
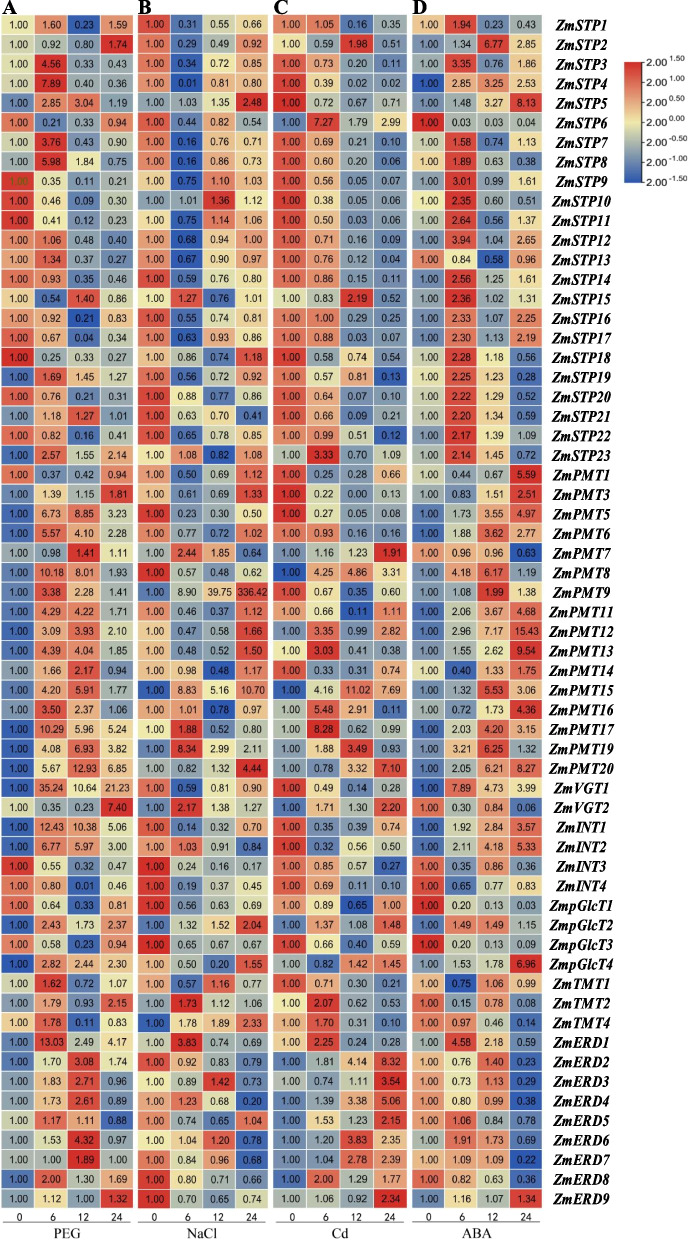
Expression analysis of *ZmMST* genes in response to abiotic stresses and exogenous ABA. **A** The expression of *ZmMST* genes at 0, 6, 12, and 24 h under drought treatment. **B** The expression of *ZmMST* genes at 0, 6, 12, and 24 h under NaCl treatment. **C** The expression of *ZmMST* genes at 0, 6, 12, and 24 h under Cd treatment. **D** The expression of *ZmMST* genes at 0, 6, 12, and 24 h under ABA treatment. For each *ZmMST* genes, three biological replicates were used to calculated the mean values ± SD (standard deviation) with the 2 − ^ΔΔCT^ method. A continuous color gradient scale is indicative of the expression level (red represents induced expression; blue represents repressed expression). The 0 h relative expression level of each treatment was taken as 1

To further analyze the function of MST family members in response to abiotic stresses, the expression patterns of MST family genes were detected in maize under abiotic stresses and ABA treatment (Fig. 7 and Additional file 11). Under drought treatment, the expression levels of most MST members were increased, especially in the PMT, ERD, and VGT subfamilies. Under NaCl treatment, the expression of only five genes was induced, of which *ZmPMT9* was the most significantly increased. Under Cd stress, the transcript levels of *ZmPMT8*/12/13/*15*/16/17/19/*20* and *ZmERD2*/*3*/*4*/*6* were significantly increased. Abscisic acid is a phytohormone involved in regulating plant responses to abiotic stresses. The expression of most MST members was induced under exogenous ABA treatment. As compared to treatment at 0 h, the relative expression of *ZmPMT9* was highest at 12 h of salt treatment and 24 h of cadmium treatment, and lowest at 6 h of salt treatment and 24 h of cadmium treatment for *ZmSTP4*. Overall, the expression of 61 *ZmMST* genes showed different alterations under the abiotic stresses and exogenous ABA treatment, and some genes were affected by multiple treatments (Additional file 12).

**Fig. 8:**
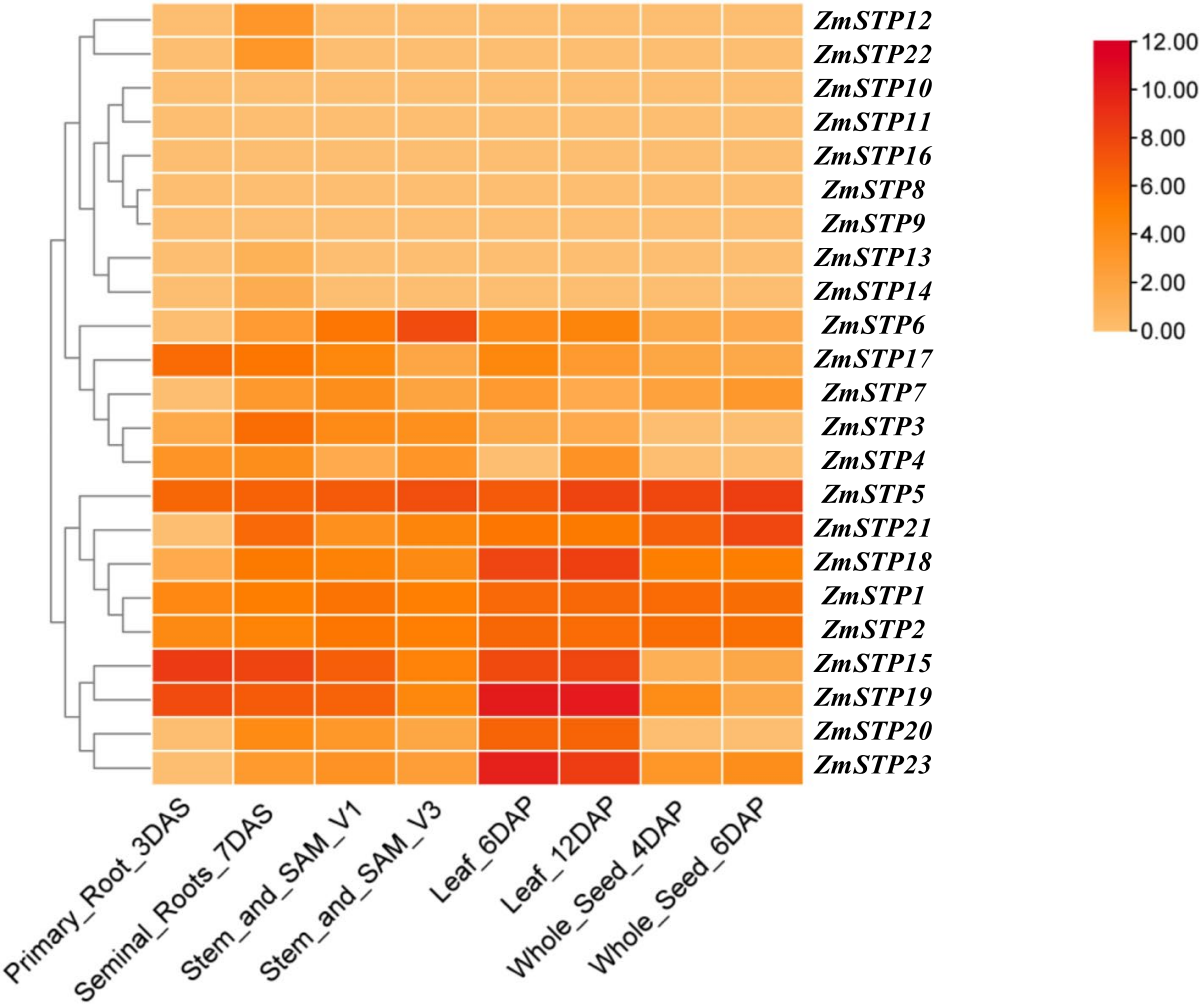
Expression profiles of *ZmSTP* genes in different tissues. Log_2_-based fold change data were used to create the heatmap. Fold changes in gene expression are indicated by the color scale. DAS: Days of growth after sprouting. DAP: Days after pollination

The expression of *MST* genes in various tissues, including roots, stems, leaves, and seeds, was also examined (Fig. 8, Additional files 13 and 14). *ZmSTP1*, *ZmSTP2*, and *ZmSTP5* were relatively high and stable in various tissues and periods, while the expression levels of *ZmSTP8*/*9*/*10*/*11*/*12*/*13*/*14*/*16* and *22* were relatively low in various tissues. However, with the development process, the expression of *ZmSTP2* in roots, stems, leaves, and seeds were rapidly up-regulated, while *ZmSTP5* showed a trend of high expression in all tissues and stages. The expression patterns of members of the same subfamily were complementary. For example, *ZmSTP19* and *ZmSTP23* were highly expressed in leaves but almost not in seeds. *ZmSTP23* was highly expressed in leaves, but expressed at low levels in roots, stems, and seeds. *ZmPMT1* and *ZmPMT13* also had similar expression patterns. According to these results, *ZmMST* genes played a variety of roles in maize growth and development.

## Discussion

Since monosaccharides such as glucose and fructose are essential for metabolism, storage, and transport, MST is crucial to the processes of carbon partitioning and abiotic stress response in plants [12, 26, 29]. Genome-wide analysis of the MST gene family has been widely carried out in many species. Fifty-three, sixty-four, and sixty-nine genes have been identified in Arabidopsis, rice, and sorghum respectively [26, 29]. However, the MST gene family has not been identified in maize. In this study, 66 *MST* genes were identified in the maize genome, and 77 MST family genes were identified in millet (Table 1 and Additional file 1). By the phylogenetic tree results, *ZmMST* genes were further divided into 7 subfamilies [30]. Each subfamily of MST was shown to be specific in the differentiation and evolution of the different Gramineae based on our findings that the number of genes in each subfamily of MST varied in maize, rice, sorghum, and millet. It was reported that the STP subfamily is the largest subfamily in rice. The ERD subfamily is the largest subfamily in Arabidopsis [26]. In maize, the largest subfamily is also the STP family, which indicates that the MST family has species-specific subfamily expansion in different plants. These expansions may be caused by gene duplication events, which may play a key role in the evolution of the MST gene family.

The previous study proved that most genes in the Arabidopsis genome were produced by species-specific expansion of the gene family [31]. Maize underwent three genome-wide replication events occurred, including approximately 110 million years ago before the differentiation of monocotyledons and dicotyledons, before the emergence of Gramineae 50 million years ago, and the genome-wide replication event after the differentiation of maize and sorghum 12 million years ago [32, 33]. Three ways of gene family expansion and doubling were found: whole-genome duplication (WGD), tandem duplication (TD), and segmental duplication (SD) [34]. WGD is a massive chromosome doubling event that increases the dose of all genes of a species at once, resulting in a large number of chromosomally doubled segments retained in the genome. Tandem duplication occurs frequently in chromosomal recombination domains, where members of tandemly duplicated gene families are typically tightly aligned on the same chromosome, forming a cluster of genes with related sequences and functions [35]. Segmental duplication occurs when duplicated genes are distant or even located on different chromosomes. In this study, multiple gene replication events were identified, including eight pairs of tandem replication gene pairs (*ZmSTP5*/*6*, *ZmSTP21*/*22*, *ZmPMT5*/*6*, *ZmPMT11*/*12*, *ZmPMT12*/*13*, *ZmPMT15*/*14*, *ZmPMT15*/*16*, and *ZmERD3*/*4*) with highly similar sequences in adjacent positions of chromosomes and twelve pairs of segmental duplication genes (*ZmSTP12*/*5*, *ZmSTP5*/*14*, *ZmSTP8*/*17*, *ZmSTP14*/*21*, *ZmSTP12*/*14*, *ZmSTP12*/*21*, *ZmPMT8*/*14*, *ZmPMT14*/*3*, *ZmPMT1*/*18*, *ZmpGlcT1*/*4*, *ZmERD2*/*5*, *ZmERD6*/*3*). The results showed that gene tandem duplication and chromosome segmental duplication are the main forms of monosaccharide transporter replication in maize.

Gene family expansion and doubling can provide new adaptability for plant growth and development to resist biotic and abiotic stress, leading to gene functional diversity, and affecting the evolution process of species [36]. Ka/Ks analysis was used to determine the relative divergence time and whether the functional differentiation of replication genes was subject to selection pressure. According to previous studies, Ka >> Ks or Ka/Ks >> 1, Ka = Ks or Ka/Ks = 1, Ka/Ks or Ka/Ks << 1, and Ka/Ks or Ka/Ks << 1 denoted that the gene was susceptible to positive selection, neutral evolution, and purifying selection, respectively [37, 38]. In this study, the Ka/Ks of all the duplicated gene pairs were calculated, and most of them showed less than one, indicating that they were subjected to purifying selection in the process of evolution. Five gene pairs subjected to strong positive selection were *ZmSTP5*/*ZmSTP12*, *ZmERD2*/*ZmERD5*, and *ZmERD3*/*ZmERD6* for segmental duplication and *ZmSTP5*/*ZmSTP6* and *ZmSTP3*/*ZmSTP4* for tandem duplication*.* This indicated that they were positively selected and rapidly evolved genes in a short period, and gene functions may have diverged. Of these three gene pairs belong to the ERD subfamily, suggesting that the ERD subfamily may be more important for maize to respond to environmental change. Synteny analysis was performed to analyze the expansion of the MST family between species. There were 47, 41, and 33 collinear gene pairs identified between maize and sorghum, foxtail millet and rice, respectively. Only one collinear pair between maize and alfalfa (*ZmpGlcT4*/*AES80568*) and *ZmpGlcT4* also formed collinear pairs with *SORBI_3003G084000* and *Os01g0133400* between maize and sorghum, rice, respectively. These results showed a closer evolutionary relationship between the two species containing more collinear gene pairs, and most of the no-collinear genes may be produced in earlier replication events.

Gene structure and conserved motif analyses were performed to further explore the evolutionary relationship in the MST family of maize. *MST* genes could be divided into seven subfamilies, and the PMT, STP, and TMT subfamilies contained fewer exons and simpler gene structures, while the pGlcT, ERD, INT, and VGT subfamilies had more exons and more complex gene structures, and gene structures were conserved in the same subfamily (Fig. 3). Motifs 1–6 were present in almost all MST family members and were vital for transport function and membrane localization, suggesting that these motifs were highly conserved domains during the evolution of the MST family. Other motifs existed in different subfamilies, indicating that these subfamily members transport different substrates. In addition, STP subfamily members contained all motifs except for motif 11, which implied a broader transport capability for different substrates. Only STP subfamily include motif 13 and 14. Miraculously, motif 10 and motif 14 only appear together in STP subfamily. The motif 7 is absent from ERD and TMT subfamilies. Only ERD and STP subfamilies have no motif11 among the MST subfamilies. The effect of genes on plants depends not only on the function of genes themselves, but also on the regulation of gene expression. The *cis*-elements in the promoter region were involved in the regulation of the gene expression, and therefore the *cis*-element analysis was important for the preliminary prediction of gene expression. The type and number of *cis*-elements in promoters of MST family genes were identified in this study. The ABA response element ABRE belongs to the phytohormone response element and exists in most MST member promoter regions, meaning that the expression of MST members might be involved in the ABA signaling pathway. Additionally, many stress response elements were also found in the promoter region of MST members, such as DRE, MYB, and MYC, suggesting that MST family genes have an important role in maize response to environmental stresses.

To further investigate the functional response to environmental stresses, the expression mode of MST family genes in the maize seedling stage was detected under different stress treatments. The expression of half of *MST* genes was significantly induced under drought treatment, while fewer genes were induced by salt and Cd stresses (Fig. 7A, B, and C). Meanwhile, the response of each subfamily in MST to different stresses was also different. Most of the PMT and ERD members responded to osmotic stress, and all ERD members were induced to expression by Cd stresses. The expression of most members was not induced in the STP subfamily under osmotic or ionic stresses. The expression patterns of MST members in response to stress treatments suggested that each subfamily might have different roles in balancing maize growth and development and responding to abiotic stress, implying that the members in each subfamily had also shown functional divergence during evolution and family expansion. ABA is a very important phytohormone in the plant response to abiotic stresses, and many stress response genes are regulated by ABA signaling [39]. In this study, the expression levels of some MST family members were induced by both ABA treatment and drought stress, indicating that these members might respond to stress in the ABA signaling pathway, while others were only induced by drought stress, meaning that they might respond to stress in the non-ABA signaling pathway.

Monosaccharide transport and distribution play critical roles in plant growth and development, as well as in response to abiotic stress [40]. Previous research indicates that *OsSTP4* functions as a transporter of fructose and mannose, as well as glucose and galactose, and responds rapidly to abiotic stresses [13, 41]. *ZmSTP2* is homologous to *OsSTP4*, responses immediately to abiotic stress in maize, thus it may play a similar role as *OsSTP4*. Salt treatment induces *ZmpGlcT2* expression, and it is the homologous gene of *OspGlcT2* and possess similar expression pattern under abiotic stresses [42]. *ZmTMT4* is induced to be expressed under salt treatment. Its rice homologous gene *OsTMT1* is studied and reports to be associated with salt stress [43]. These results may suggest that homologous genes perform the similar functions in different plants. Monosaccharide transporters impact plant growth and development, as well as abiotic stress response mechanisms, by transporting and distributing monosaccharides. The genome-wide identification and selection of genes that may be similar to previously reported genes reacting to abiotic stress will give theoretical support and insights for future investigations on maize resistance genes.

Together, we have identified maize MST family genes. Our findings could contribute to future research on maize MST family genes and provide the foundation for additional investigation of the fundamental functions of this significant monosaccharide transporter family. These findings provide insight into the possible roles of genetic improvement in the capacity of maize to respond to abiotic challenges and may be used to identify relevant candidate MST family genes for functional research.

## Conclusions

In summary, a total of 66 *ZmMST* genes were identified from maize and divided into seven subfamilies. Phylogenetic tree, synteny, and collinearity analyses provide preliminary insights into dissecting the evolution and expansion of the MST family. Meanwhile, the expression analysis provides valuable clues for exploring the potential function of MST in balancing maize growth and development and abiotic stress response. These findings will be helpful for us to deeply understand the functions of maize *MST* genes and provide some important information for functional analysis in the future.

## Methods

### Identification and evolutionary analysis

The complete amino acid and nucleotide sequences of Zea mays B73 RefGen_v4 were downloaded from MaizeGDB (https://maizegdb.org/). In addition, MaizeGDB (https://maizegdb.org/) transcriptome data were obtained. Seventy-nine different samples comprised the maize inbred line B73 RNA-seq gene map [44]. Means and standard deviations were calculated for the data files and the results were finally presented in the form of heatmaps (https://github.com/CJ-Chen/TBtools) [45]. The transcriptome information was chosen from eight distinct maize tissues and developmental stages. From Ensembl (https://asia.ensembl.org/index.html), protein sequences for the rice MST and the Arabidopsis MST were acquired. The hidden Markov model repository was built using known MST protein sequences, and HMMER (http://hmmer.org/) was used to query the maize dataset [46]. By utilizing *MST* genes from rice and Arabidopsis as queries in a BLAST search, *MST* genes from maize were investigated. Using the PFAM (http://pfam.xfam.org/) and CDD (https://www.ncbi.nlm.nih.gov/cdd/) databases, the conserved domains of the discovered *ZmMST* genes were predicted [47, 48]. Using MEGA 7.0 (https://www.megasoftware.net/) and ClustalW software (https://www.genome.jp/tools-bin/clustalw), evolutionary trees were created for the MST proteins from Arabidopsis, rice, and maize (with 1000 bootstrap replicates) [49, 50]. Using a MapChart (http://mg2c.iask.in/mg2c_v2.0/) and the chromosomal start and termination data received from MaizeGDB (https://maizegdb.org/), the chromosomal locations of *ZmMST* genes were determined [51]. Tandem duplicated genes were found using the tools for multiple covariance scanning (MCScanX, http://chibba.pgml.uga.edu/mcscan2/MCScanX.zip) [52].

### Sequence analysis

The molecular weight (MW) and isoelectric point (pI) of the ZmMST proteins were predicted using the ExPASy proteomics system (http://web.expasy.org/protparam/) (Table 1) [53]. The conserved protein motifs of *ZmMST* genes were discovered using MEME Suite (http://meme-suite.org/), and they were further annotated with TBtools (https://github.com/CJ-Chen/TBtools) [45, 54]. Fifteen motifs, with lengths ranging from 6 to 50 bp, made up the domain structure (Additional file 15). Using GSDS (http://gsds.gao-lab.org), the gene structure was evaluated [55]. The PlantCARE database (http://bioinformatics.psb.ugent.be/webtools/plantcare/html/) was used to predict the 1500 bp sequence upstream of the *cis*-acting components of the coding sequences (Additional file 16). Further examination was performed on the components (ABRE, DRE, LTRE, ERE, and MBS) connected to the abiotic stress response [56, 57].

### Replication events and Ka/Ks analysis of MST genes

MCScanX (http://chibba.pgml.uga.edu/mcscan2/MCScanX.zip) was used to analyze correlations between *ZmMST* genes and single or multiple intergenomic variables as well as associations within genomes [58]. Gene family expansion and doubling can occur through several different processes, including whole-genome duplication or polyploidization, tandem duplication, segmental duplication, transposon-mediated transposon duplication, and retro-position. Finally, a graph of the intragenomic duplication events and gene density findings was created using TBtools software (https://github.com/CJ-Chen/TBtools) (Fig. 4).

ClustalW (https://www.genome.jp/tools-bin/clustalw) was used to determine the ratio of Ka (non-synonymous substitution rate) and Ks (synonymous substitution rate) to investigate the selection pressure on the ZmMST family. The time of occurrence of segmental duplication events for homologous genes was calculated as T = Ks/2λ × 10^−6^, where λ is the rate of molecular substitution in grasses (6.5 × 10^−9^), and expressed as a million years ago (Mya) [59].

### Plant treatments and quantitative real-time PCR analysis

The autogamous maize cultivar “inbred line” was used in the study. Seeds were preserved in our laboratory and incubated in the seedling culture room of the Laboratory of Plant Physiology and Germplasm, Shenyang Agricultural University. The seeds were disinfected with 75% ethanol and washed with distilled water after 1 min to remove the residual ethanol. The cleaned seeds were evenly sown in seedling pots and irrigated with distilled water to allow the vermiculite to absorb sufficient water. Hoagland’s nutrient solution (pH 6.0) was added to the basal tray in which the seedling pots were placed to ensure that the roots could access the nutrient solution. The nutrient solution was replaced every 3 days until the seedlings attained the three-leaf stage [60]. The three-leaf seedlings were then treated with drought, salt, Cd stresses, and exogenous ABA by application of half-strength Hoagland’s nutrient solution supplemented with 20% PEG for drought stress treatment, 200 mmol/L NaCl for salt stress treatment, 40 mg/L CdCl_2_ for Cd stress treatment, and 100 µmol/L ABA for ABA stress treatment [61]. The uppermost mature leaves were collected at 0, 6, 12, and 24 h after initiation of the stress treatment, with three biological replicates at each time point. The tested leaves were immediately frozen in liquid nitrogen and kept at -80 °C.

Total RNA isolation and quantitative real-time PCR (qRT-PCR) analysis were performed to analyze the expression of maize genes under salt, drought, Cd stress and exogenous ABA treatment [62]. A total of 61 maize *MST* genes were used for the analysis. Total RNA from plant leaves was extracted using TRIzol Reagent (CW Biotech) and subjected to DNase I treatment to remove genomic DNA contamination. The RNA concentration was determined utilizing a BioDrop ultramicro ultraviolet nucleic acid assay. First-strand cDNA was synthesized from 1 µg of total RNA using the UEIris II RT-PCR system. qRT-PCR assays were performed using a real-time PCR analyzer (Bio-Rad, Applied Biosystems PCR, SCILOGEX Gradient Thermal Cycler PCR Instrument TC1000-G). Each reaction mixture contained 10 µL of 2 × SYBR® Green Pro Taq HS Premix, 1.0 µL cDNA sample, 0.4 µL forward primer (final concentration 10 µM), and 0.4 µL reverse primer (final concentration 10 µM) in a final volume of 20 µL. The thermal-cycling protocol was as follows: 95 °C for 5 min, then 45 cycles of 95 °C for 15 s and 60 °C for 1 min. Melting curve analysis was used to verify the specificity of the reaction. Three technical replicates of each cDNA sample were analyzed. The *Zm00001d013367* genes were selected as an internal control to normalize the transcript levels of *ZmMST* genes [63]. The relative gene expression levels were calculated using the 2^−∆∆CT^ method and analyzed for significance using SPSS 20 (https://spssau.com/) (Additional file 11 and 17). The normalized data were processed with TBtools and plotted as a heatmap to visualize the changes in MST gene expression (https://github.com/CJ-Chen/TBtools) [64]. Row-scale and log-scale normalization calculations and row clustering were performed on the heatmaps. The number of genes with relative gene expression greater than 2 and relative gene expression less than 0.5 under the four treatments were shown by using the online site Venny 2.1.0 (https://bioinfogp.cnb.csic.es/tools/venny/) to create Venn diagrams. All primer pairs were designed with Primer (v5.0) software (http://www.broadinstitute.org/ftp/pub/software/Primer5.0/) and were listed in Additional file 18.

### Supplementary Information

Supplementary Material 1.

Supplementary Material 2.

Supplementary Material 3.

Supplementary Material 4.

Supplementary Material 5.

Supplementary Material 6.

Supplementary Material 7.

Supplementary Material 8.

Supplementary Material 9.

Supplementary Material 10.

Supplementary Material 11.

Supplementary Material 12.

Supplementary Material 13.

Supplementary Material 14.

Supplementary Material 15.

Supplementary Material 16.

Supplementary Material 17.

Supplementary Material 18.

## Data Availability

All data analyzed in this study are included in this published article and its Additional File. Raw transcriptomic reads are available from NCBI via the following BioProject IDs: PRJNA171684, PRJEB10574, PRJNA226757, PRJNA244661, PRJNA323555, and PRJNA369690. The datasets analyzed in this study are available in the Ensembl (https://plants.ensembl.org/index.html) and Transcriptome Data (http://maize.plantbiology.msu.edu/MSU_func_download.shtml).

## Abbreviations

MST: Monosaccharide transporter
MFS: Major facilitator superfamily
SUT: Sucrose transporter
STP: Sugar Transport Protein
PMT: Polyol/Monosaccharide Transporter
VGT: Vacuolar Glucose Transporter
INT: Inositol Transporter
pGlcT: Plastidic Glucose Transporter
TMT: Tonoplast Membrane Transporter
ERD: Early-Responsive to Dehydration six-like
chr: Chromosome
WGD: Whole-genome duplication
MW: Molecular weight
pI: Isoelectric point
Ka: Non-synonymous substitution rate
Ks: Synonymous substitution rate
Mya: Million years ago
qRT-PCR: Quantitative real-time PCR
ABA: Abscisic Acid
SA: Salicylic acid
MeJA: Methyl jasmonate
