# Supplementary figures and images for: Genome-wide identification and investigation of monosaccharide transporter gene family based on their evolution and expression analysis under abiotic stress and hormone treatments in maize (Zea mays L.)
Source: BMC Plant Biol. 2024 Jun 4;24:496. doi: 10.1186/s12870-024-05186-2 (PMC11149190; doi:10.1186/s12870-024-05186-2)

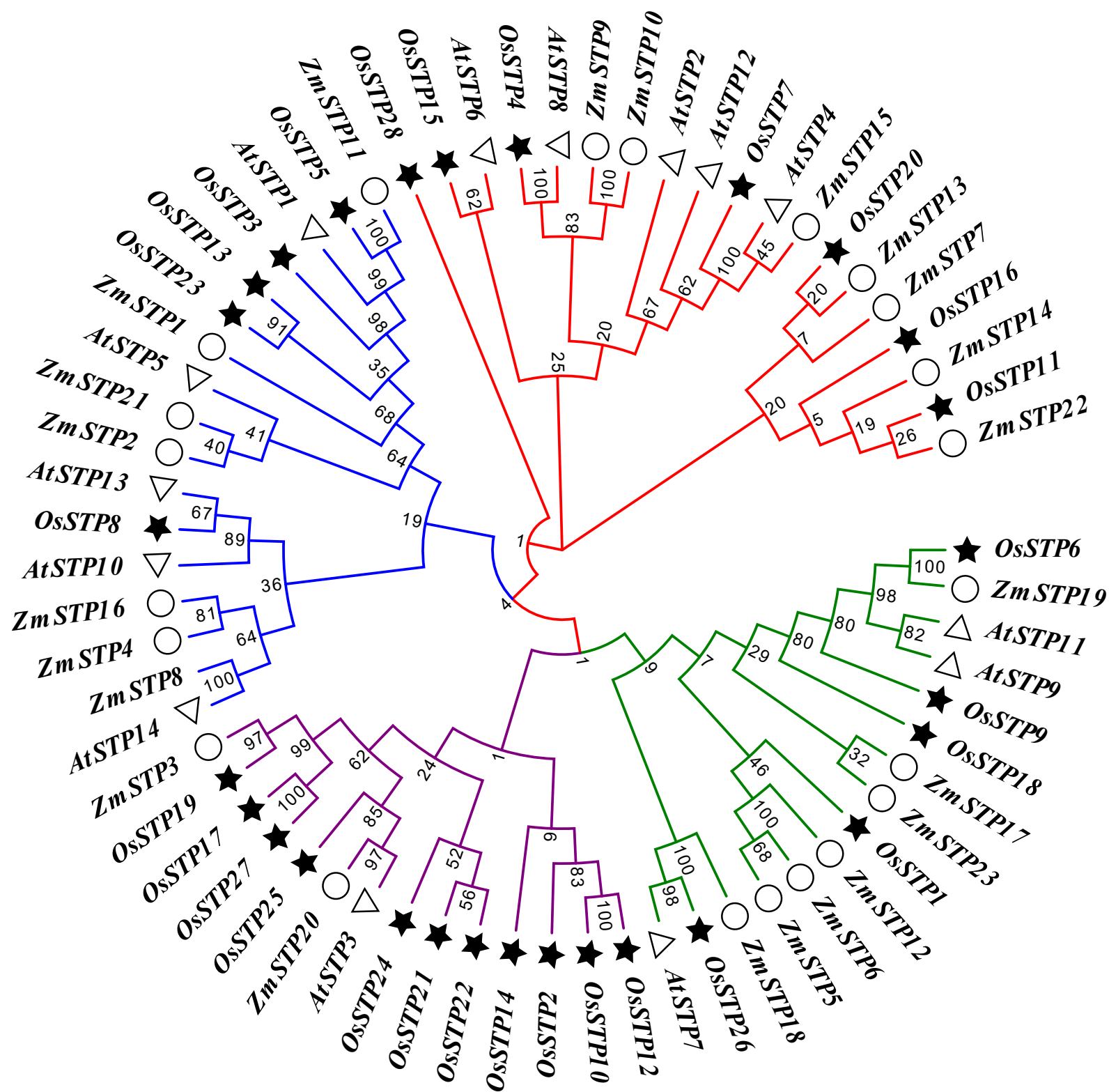

**Fig. S1:** Phylogenetic tree for STP proteins of maize, rice, and Arabidopsis.

Supplement: Supplementary file 2 — Supplementary Material 2. [file 12870_2024_5186_MOESM2_ESM.pdf]

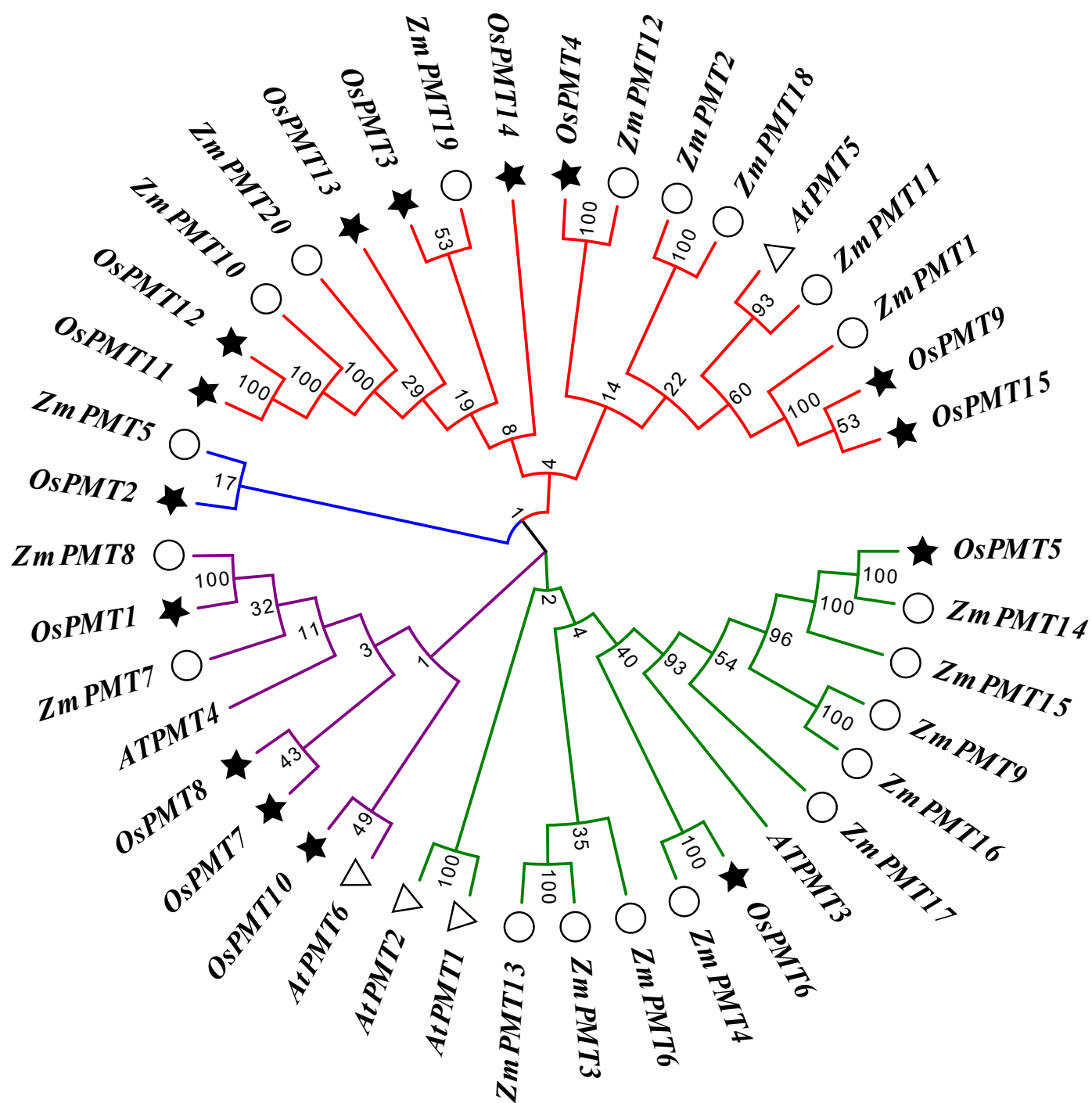

**Fig. S2:** Phylogenetic tree for PMT proteins of maize, rice, and Arabidopsis.

Supplement: Supplementary file 3 — Supplementary Material 3. [file 12870_2024_5186_MOESM3_ESM.pdf]

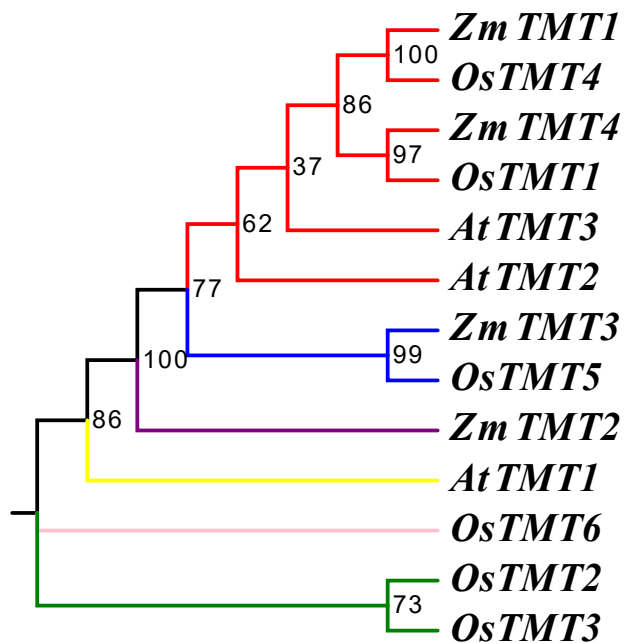

**Fig. S3:** Phylogenetic tree for TMT proteins of maize, rice, and Arabidopsis.

Supplement: Supplementary file 4 — Supplementary Material 4. [file 12870_2024_5186_MOESM4_ESM.pdf]

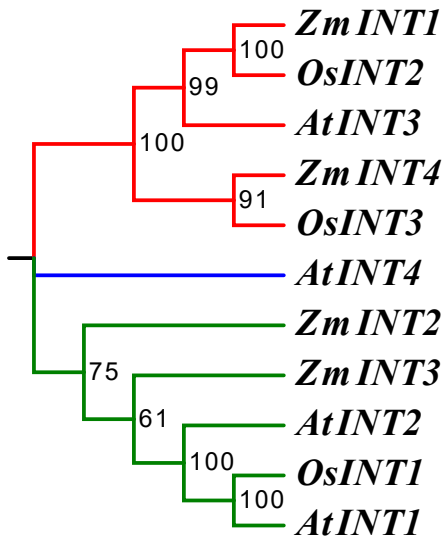

**Fig. S4:** Phylogenetic tree for INT proteins of maize, rice, and Arabidopsis.

Supplement: Supplementary file 5 — Supplementary Material 5. [file 12870_2024_5186_MOESM5_ESM.pdf]

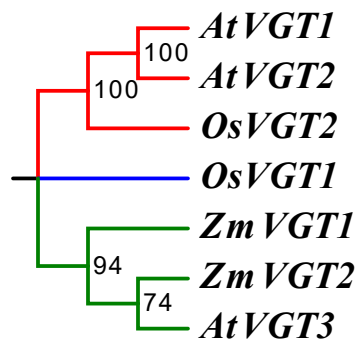

**Fig. S5:** Phylogenetic tree for VGT proteins of maize, rice, and Arabidopsis.

Supplement: Supplementary file 6 — Supplementary Material 6. [file 12870_2024_5186_MOESM6_ESM.pdf]

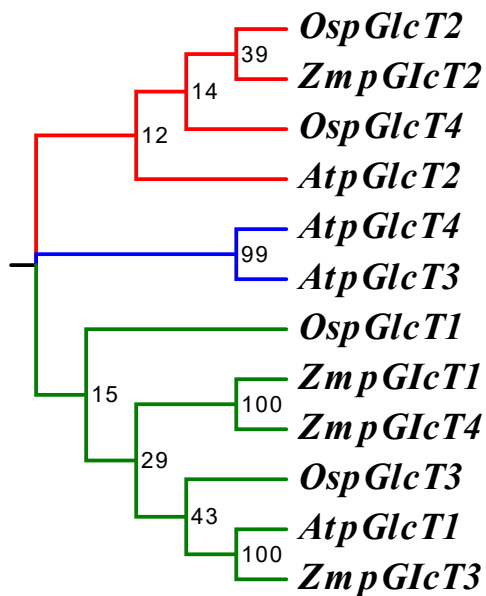

**Fig. S6:** Phylogenetic tree for pGlcT proteins of maize, rice, and Arabidopsis.

Supplement: Supplementary file 7 — Supplementary Material 7. [file 12870_2024_5186_MOESM7_ESM.pdf]

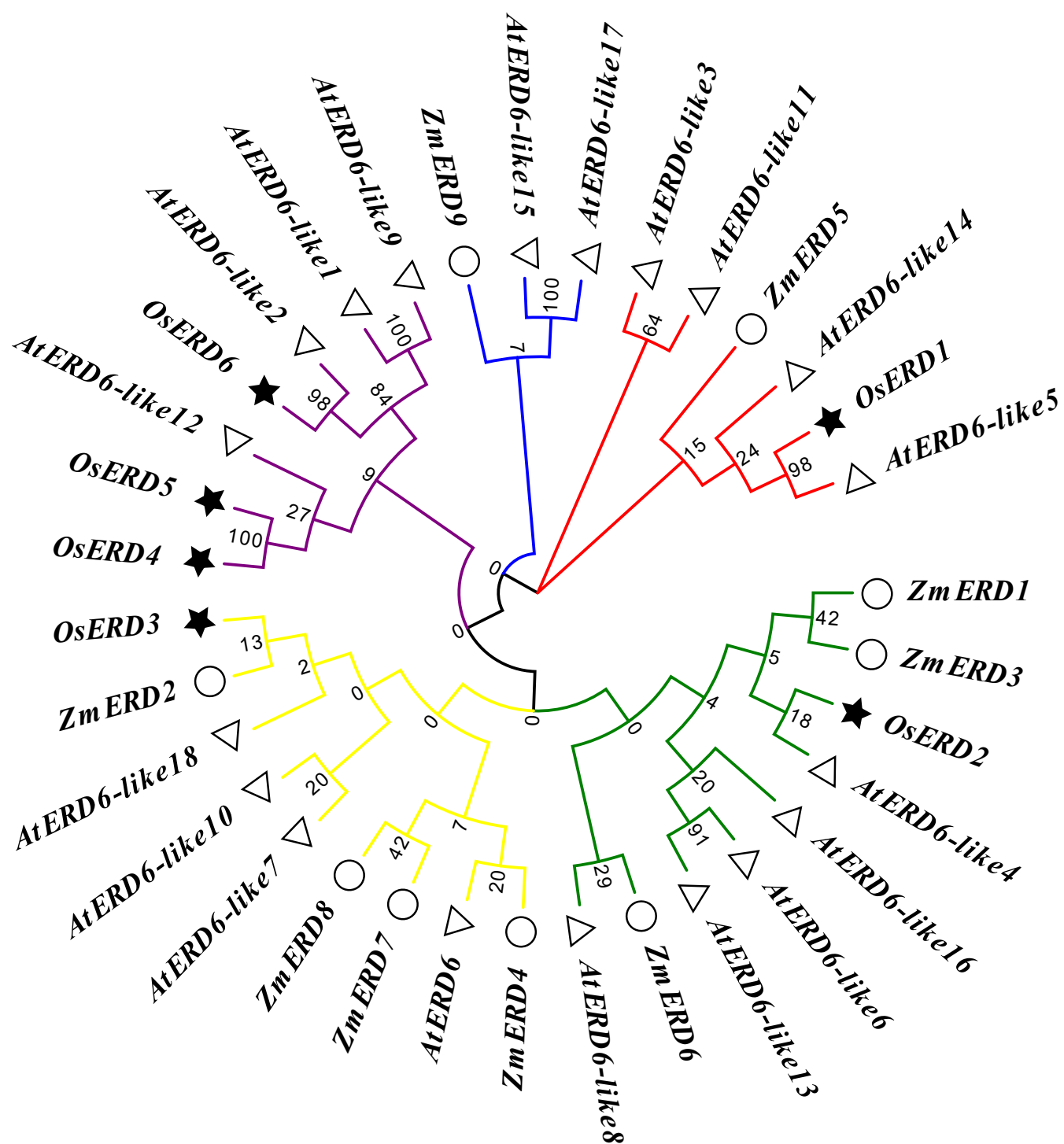

**Fig. S7:** Phylogenetic tree for ERD proteins of maize, rice, and Arabidopsis.

Supplement: Supplementary file 8 — Supplementary Material 8. [file 12870_2024_5186_MOESM8_ESM.pdf]

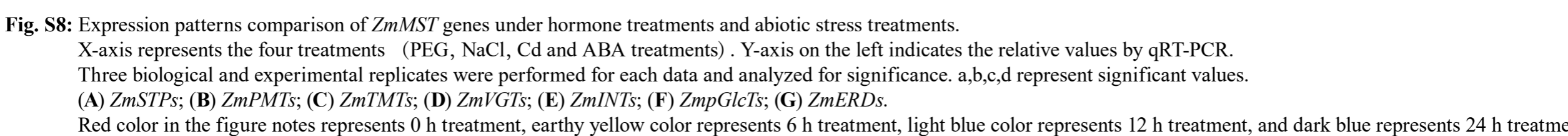

Supplement: Supplementary file 11 — Supplementary Material 11. [file 12870_2024_5186_MOESM11_ESM.pdf]

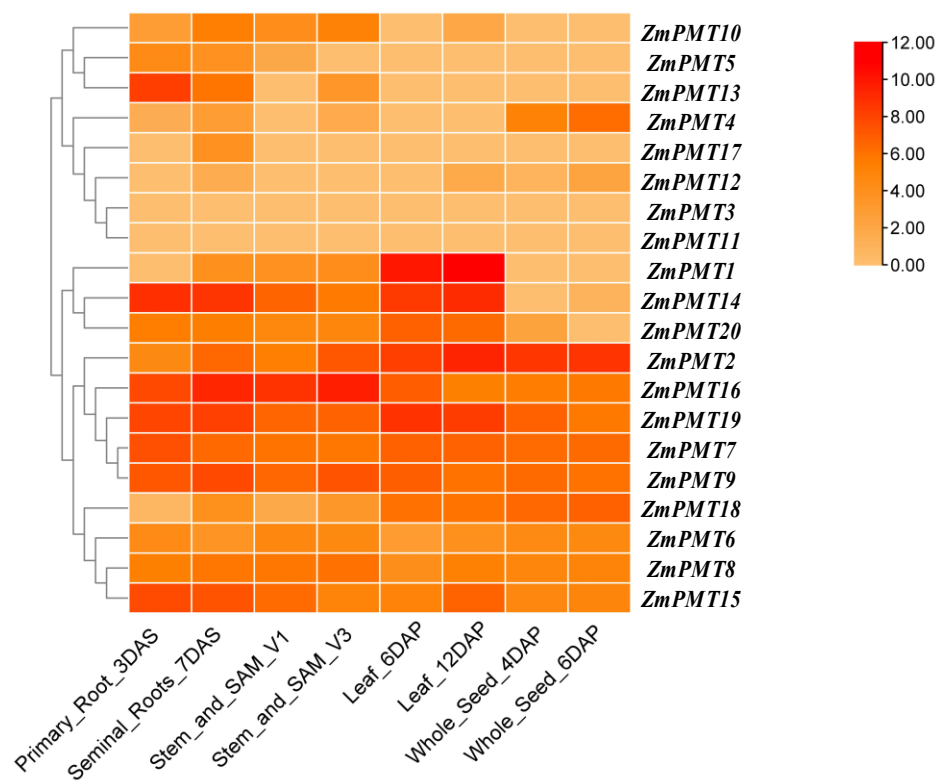

**Fig. S10:** Expression profiles of *ZmPMT* genes in different

Supplement: Supplementary file 13 — Supplementary Material 13. [file 12870_2024_5186_MOESM13_ESM.pdf]
