# Supplementary material for: Genome-wide identification and investigation of monosaccharide transporter gene family based on their evolution and expression analysis under abiotic stress and hormone treatments in maize (Zea mays L.)
Source: BMC Plant Biol. 2024 Jun 4;24:496. doi: 10.1186/s12870-024-05186-2 (PMC11149190; doi:10.1186/s12870-024-05186-2)
Supplement: Supplementary file 12 — Supplementary Material 12. [file 12870_2024_5186_MOESM12_ESM.pdf]

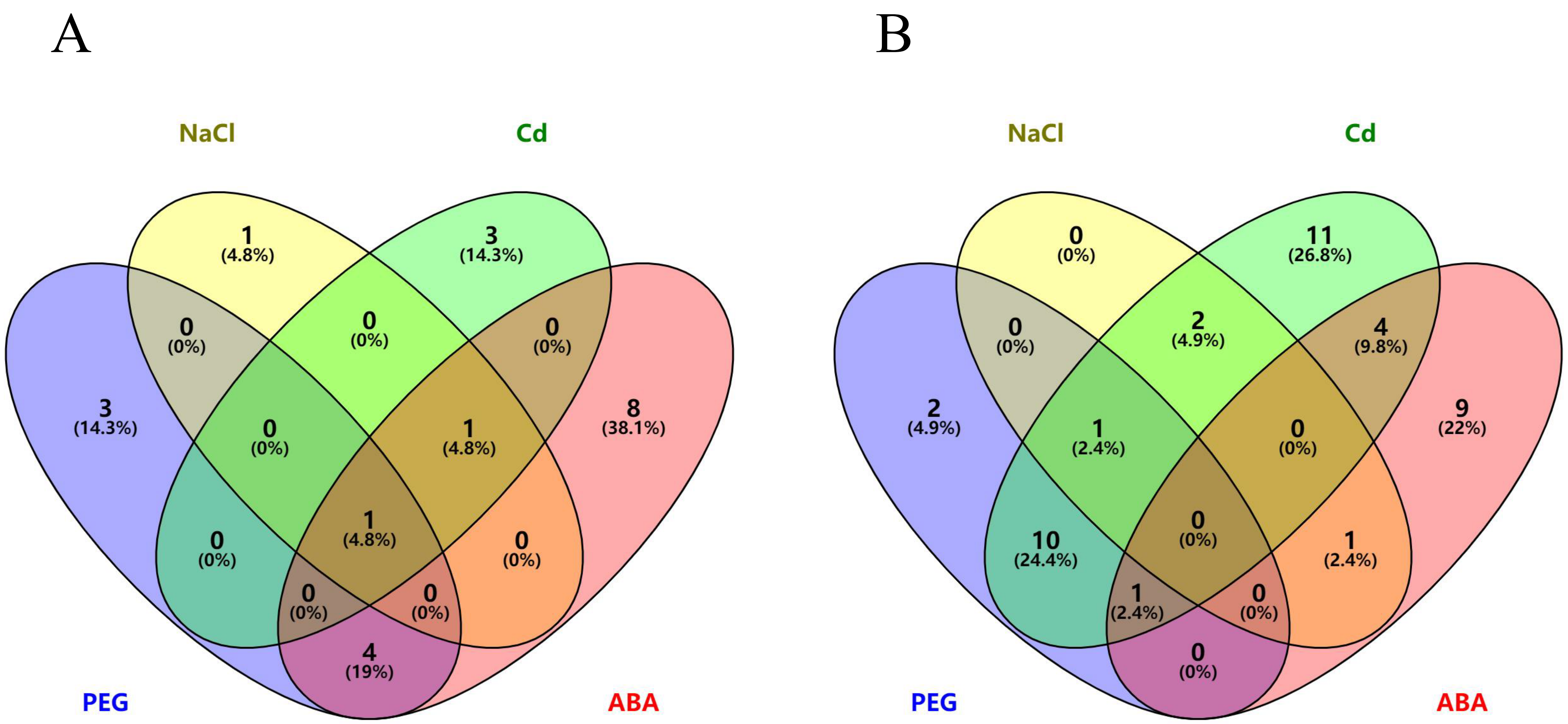

Fig. S9: Venn diagram of the relative ZmMST genes expression under four treatments. **A** The number of ZmMST genes is induced under four treatments. **B** The number of ZmMST genes is inhibited under the four treatments. Blue oval-shaped,
