# Supplementary material for: Genome-wide identification and investigation of monosaccharide transporter gene family based on their evolution and expression analysis under abiotic stress and hormone treatments in maize (Zea mays L.)
Source: BMC Plant Biol. 2024 Jun 4;24:496. doi: 10.1186/s12870-024-05186-2 (PMC11149190; doi:10.1186/s12870-024-05186-2)
Supplement: Supplementary file 14 — Supplementary Material 14. [file 12870_2024_5186_MOESM14_ESM.pdf]

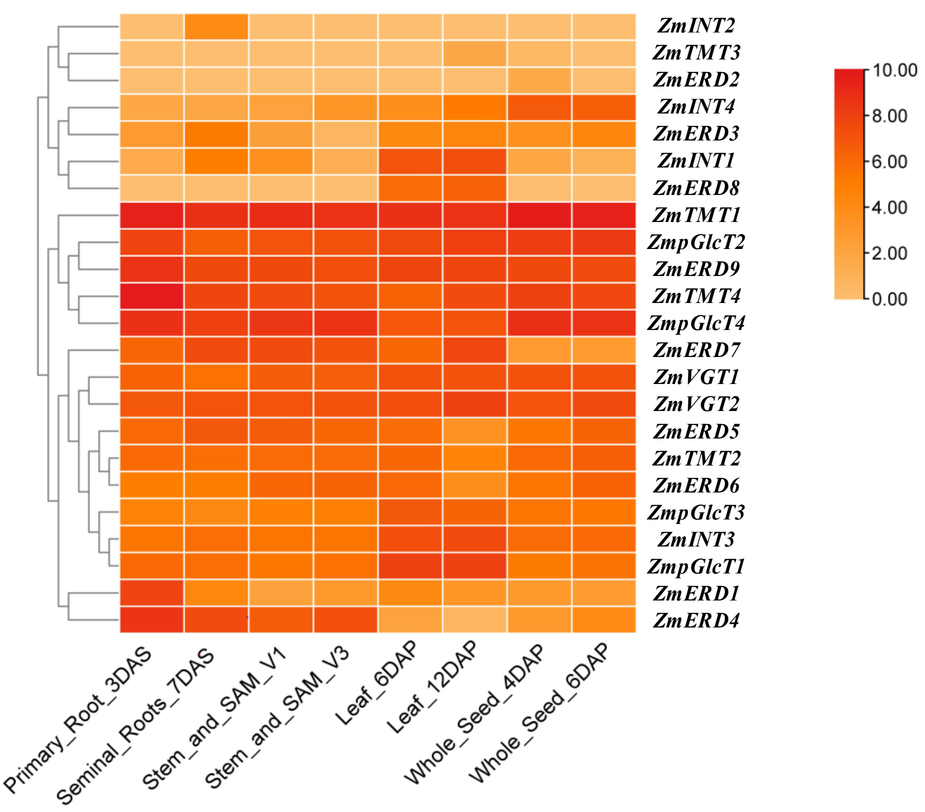

Fig. S11: Expression profiles of *ZmTMT* genes, *ZmINT* genes, *ZmVGT* genes, *ZmpGlcT* genes, and *ZmERD* genes in different tissues.
